# Detection of vancomycin-resistant *Enterococcus faecium* hospital-adapted lineages in municipal wastewater treatment plants indicates widespread distribution and release into the environment

**DOI:** 10.1101/gr.232629.117

**Published:** 2019-04

**Authors:** Theodore Gouliouris, Kathy E. Raven, Danesh Moradigaravand, Catherine Ludden, Francesc Coll, Beth Blane, Plamena Naydenova, Carolyne Horner, Nicholas M. Brown, Jukka Corander, Direk Limmathurotsakul, Julian Parkhill, Sharon J. Peacock

**Affiliations:** 1 Department of Medicine, University of Cambridge, Cambridge CB2 0QQ, United Kingdom;; 2 Public Health England, Clinical Microbiology and Public Health Laboratory, Addenbrooke's Hospital, Cambridge CB2 0QW, United Kingdom;; 3 Wellcome Sanger Institute, Hinxton, Cambridge CB10 1SA, United Kingdom;; 4 London School of Hygiene and Tropical Medicine, London WC1E 7HT, United Kingdom;; 5 British Society for Antimicrobial Chemotherapy, Birmingham B1 3NJ, United Kingdom;; 6 Institute of Basic Medical Sciences, University of Oslo, 0372 Oslo, Norway;; 7 Mahidol-Oxford Tropical Medicine Research Unit, Mahidol University, Bangkok, 10400, Thailand

## Abstract

Vancomycin-resistant *Enterococcus faecium* (VREfm) is a leading cause of healthcare-associated infection. Reservoirs of VREfm are largely assumed to be nosocomial although there is a paucity of data on alternative sources. Here, we describe an integrated epidemiological and genomic analysis of *E. faecium* associated with bloodstream infection and isolated from wastewater. Treated and untreated wastewater from 20 municipal treatment plants in the East of England, United Kingdom was obtained and cultured to isolate *E. faecium*, ampicillin-resistant *E. faecium* (AREfm), and VREfm. VREfm was isolated from all 20 treatment plants and was released into the environment by 17/20 plants, the exceptions using terminal ultraviolet light disinfection. Median log_10_ counts of AREfm and VREfm in untreated wastewater from 10 plants in direct receipt of hospital sewage were significantly higher than 10 plants that were not. We sequenced and compared the genomes of 423 isolates from wastewater with 187 isolates associated with bloodstream infection at five hospitals in the East of England. Among 481 *E. faecium* isolates belonging to the hospital-adapted clade, we observed genetic intermixing between wastewater and bloodstream infection, with highly related isolates shared between a major teaching hospital in the East of England and 9/20 plants. We detected 28 antibiotic resistance genes in the hospital-adapted clade, of which 23 were represented in bloodstream, hospital sewage, and municipal wastewater isolates. We conclude that our findings are consistent with widespread distribution of hospital-adapted VREfm beyond acute healthcare settings with extensive release of VREfm into the environment in the East of England.

Vancomycin-resistant *Enterococcus faecium* (VREfm) is a leading cause of healthcare-associated infection and particularly affects critically ill and immunocompromised patients (Sievert et al. 2013). This problem has been driven by the emergence and global dissemination of successful lineages belonging to the hospital-adapted *E. faecium* clade designated as A1 (previously known as clonal complex 17 [CC17] by multilocus sequencing typing [MLST]) (Willems et al. 2005; Arias and Murray 2013; Lebreton et al. 2013; Guzman Prieto et al. 2016). Clade A1 isolates are characterized by chromosomally mediated ampicillin and fluoroquinolone resistance, high genomic plasticity, and the accumulation of horizontally acquired genes encoding virulence factors and antibiotic resistance (Willems et al. 2001; Leavis et al. 2004, 2006; Hegstad et al. 2010; Palmer et al. 2010). This includes resistance to vancomycin, the drug of choice for ampicillin-resistant *E. faecium* (AREfm) infection (Howden et al. 2013; Raven et al. 2017). In contrast to clade A1, an animal-associated clade A2 and a distantly related human commensal (or community-associated) clade B are rarely associated with invasive infection or vancomycin resistance (Lebreton et al. 2013; Raven et al. 2016).

Carriage of nosocomial pathogens precedes infection, and effective strategies to prevent infection are built on an understanding of how and from where people acquire their infecting organism. Genomic studies of nosocomial *E. faecium* infection have confirmed that transmission of *E. faecium* clade A1 is common (Howden et al. 2013; Pinholt et al. 2017; Raven et al. 2017; van Hal et al. 2017). A key unknown, however, is whether the development of carrier status is solely attributable to acquisition in hospitals or whether external reservoirs in the wider community and the environment could contribute to the background carriage rate. There is also a lack of information regarding rates of VREfm carriage in community populations, including those receiving healthcare and otherwise healthy populations. Municipal wastewater represents a surrogate reservoir containing pooled bacteria from human populations, the geographical spacing of which means that these receive waste from a differing case-mix, including plants that directly receive hospital waste and those that do not, in addition to being located in urban and rural settings (Cai et al. 2014).

Here, we describe a study that aimed to use wastewater to generate indirect evidence for the extent to which healthcare-associated *E. faecium* is disseminated in the community. This took an integrated approach that combined microbiological, epidemiological, and bacterial whole-genome sequence data and compared the genetic relatedness and presence of antibiotic resistance and putative virulence genes in *E. faecium* from sewage and patients with bloodstream infection in the same geographic region.

## Results

### Isolation of *E. faecium* from wastewater

**Figure 1.**
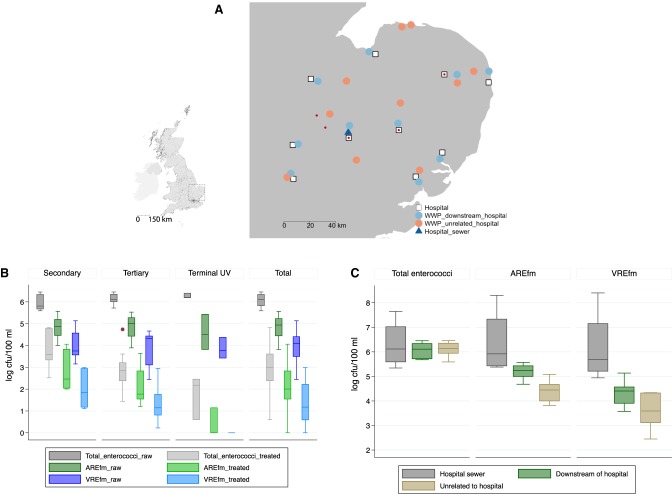
Geographic origin of *E. faecium* isolates and wastewater counts. (*A*) Map of 20 wastewater treatment plants in the East of England tested for *E. faecium*, 10 of which were directly downstream from acute hospitals belonging to the Cambridge University Hospitals NHS Foundation Trust (CUH) referral network. (WWP) Wastewater plant. Red dots indicate the source of bloodstream isolates. (*Inset*) Map of the United Kingdom, with square denoting the East of England. (*B*) Log_10_ counts of *E. faecium* recovered from 100 mL of wastewater under increasing antibiotic selection for untreated and treated wastewater according to water treatment type: secondary (*n* = 7), tertiary (*n* = 10), UV light treatment (*n* = 3), and for all plants (*n* = 20). All comparisons between treated and untreated wastewater counts were statistically significant (*P* < 0.001), as were comparisons of count reductions according to treatment type for each of total enterococci, AREfm, and VREfm (*P* = 0.02, *P* < 0.001, and *P* = 0.001, respectively). (*C*) Median log_10_ counts of *E. faecium* recovered from 100 mL of wastewater under increasing antibiotic selection from untreated wastewater from the main CUH sewer on four separate occasions, from 10 wastewater plants located downstream from acute hospitals, and from 10 wastewater plants unrelated to acute hospital waste. Boxes represent interquartile ranges, whiskers 1.5 times the interquartile range, and dots outside values (*B*,*C*).

A cross-sectional survey was conducted between June 2014 and January 2015 to isolate and quantify *E. faecium* in untreated and treated wastewater obtained from 20 municipal treatment plants in the East of England, half of which were not in direct receipt of hospital waste (Fig. 1A). Treated samples were obtained at the point of release into surface waters. Ampicillin-resistant *E. faecium* (a surrogate marker for hospital-adapted lineages) and VREfm were isolated from untreated wastewater at all 20 plants. AREfm and VREfm were isolated from 18 and 17 treated samples, respectively, the negative samples being from plants using terminal ultraviolet light decontamination.

Wastewater treatment was associated with an average reduction in count of 3.0, 2.7, and 2.5 log_10_ cfu/100 mL for all enterococci, AREfm, and VREfm, respectively (comparison of untreated versus treated wastewater, *P* < 0.001 in all cases) (Fig. 1B). The median count of VREfm in untreated and treated wastewater was 12,463 and 14 cfu/100 mL, respectively (*P* < 0.001). The log_10_ reduction was not dependent on *E. faecium* counts in untreated wastewater but varied by treatment process. The highest level of treatment processes used at the plants was secondary (*n* = 7), tertiary (*n* = 10), or terminal ultraviolet light decontamination (*n* = 3) (see Supplemental Methods and Supplemental Table S1 for further details). Compared with secondary treatment, tertiary treatment reduced AREfm and VREfm by a further 0.8 and 0.7 log_10_ cfu/100 mL on average, respectively. UV light reduced the concentration of AREfm and VREfm by a further 2.4 and 2.0 log_10_ cfu/100 mL, respectively (*P* < 0.001 and 0.002, respectively).

Four sewage samples were also obtained for culture from the main hospital sewer at the Cambridge University Hospitals NHS Foundation Trust (CUH, a major teaching hospital in the East of England) between September 2014 and December 2015. AREfm and VREfm were isolated from all four samples. Bacterial counts on selective agar for AREfm and VREfm were roughly equivalent to the total enterococcal count on nonselective agar (median log_10_ cfu/100 mL 5.9, 5.7, and 6.1, respectively) (Fig. 1C), indicating that drug-resistant *E. faecium* predominated. This contrasted with untreated municipal wastewater, where the counts of AREfm and VREfm were 5.7% (IQR 2.9%–14.5%) and 1.1% (IQR 0.4%–2.6%) of the counts of total enterococci, respectively (Fig. 1B). Median log_10_ counts of AREfm and VREfm in 100 mL of untreated wastewater from plants that were in direct receipt of hospital sewage were significantly higher than plants that were not (5.2 vs. 4.4, *P* < 0.001, and 4.3 vs. 3.5, *P* = 0.008, respectively), although total enterococcal counts were not significantly different (6.0 vs. 6.1, *P* = 0.60) (Fig. 1C).

### Comparing the genomes of *E. faecium* from wastewater and invasive disease

**Figure 2.**
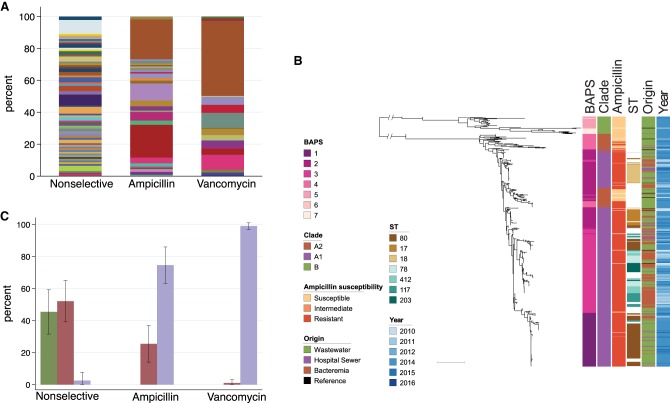
Genetic characterization of *E. faecium* isolates from wastewater and bloodstream infections. (*A*) MLST sequence types of 383 *E. faecium* isolates recovered from wastewater according to antibiotic selection during isolation. Nonselective: no selection for antibiotic resistance; ampicillin: ampicillin-resistance selection; vancomycin: vancomycin-resistance selection. (*B*) Midpoint rooted maximum-likelihood tree based on SNPs in 1336 core genes of 620 *E. faecium* (383 wastewater, 40 hospital sewage, 187 from bloodstream, and 10 reference isolates) labeled by hierarchical Bayesian cluster (1–7), clade (B, A2, and A1), ampicillin-susceptibility, commonest STs, origin, and year of isolation. Scale bar, ∼10,000 SNPs. (*C*) Proportion of each *E. faecium* clade recovered from wastewater according to antibiotic selection during isolation. A single isolate of clade A2 recovered on the vancomycin selective plate was vancomycin-susceptible. Error bars, standard deviation.

We selected 423 *E. faecium* isolates for sequencing (383 from treatment plants and 40 from the CUH sewer) (see Supplemental Methods for further details of isolation and selection). In silico MLST of the 383 isolates from treatment plants resolved 93 sequence types (STs), including 28 novel STs (including two previously nontypeable isolates due to the absence of *pstS* assigned to ST1478) (Carter et al. 2016; Raven et al. 2016), indicating a high genetic diversity (STs are listed in Supplemental Tables S2, S3). *E. faecium* from nonselective agar was the most diverse (60 STs, 92 isolates, 0.65 ST/isolate), compared with 29 STs for *E. faecium* selected for ampicillin resistance (112 isolates, 0.26 ST/isolate) and 17 STs for *E. faecium* selected for vancomycin resistance (179 isolates, 0.09 ST/isolate) (Fig. 2A; Supplemental Table S3). In silico MLST of the 40 isolates from the hospital sewer resolved 15 STs, the majority of which were either ST18 (*n* = 10) or ST80 (*n* = 10) and included four novel STs (Supplemental Table S2). These findings are consistent with specific subsets of STs being associated with drug resistance.

We then compared the genomes of the 423 wastewater *E. faecium* isolates with 187 *E. faecium* isolates associated with bloodstream infection in patients in the East of England between 2010 and 2016. Phylogenetic reconstruction using SNPs in the core genome of these 610 isolates together with 10 nonstudy genomes from open access databases demonstrated that the population divided into two major lineages, which were consistent with clade B (43 isolates), and clade A (577 isolates) (Fig. 2B). Hierarchical Bayesian clustering resolved seven BAPS (Bayesian Analysis of Population Structure) groups, of which three corresponded to clade B and four to clade A, the latter including BAPS-2 corresponding to the ST17/18 lineage, BAPS-3 corresponding to ST78, and BAPS-1 predominantly corresponding to ST80 (Fig. 2B).

BAPS-4 had several distinctive features, including genomes that resided on long branches (indicating distantly related isolates) and ampicillin-susceptible isolates (consistent with clade A2). Furthermore, this BAPS group was paraphyletic (located in two different positions in the tree). The distinction between A1 and A2 was resolved by constructing a phylogeny based on core genome mapping to the Aus0004 strain (Lam et al. 2013) after excluding the most basal A2 isolates (designated A2.1) (Supplemental Fig. S1). These findings suggested that clade A2 was heterogeneous and contained a lineage (designated here as A2.2) that resided between A2.1 and A1, including AREfm isolates belonging to STs associated previously with carriage by domestic dogs (e.g., ST19, ST192, ST266, ST401) (de Regt et al. 2012), although distinct from other ST192 isolates that clearly resided in clade A1. These findings are indicative of a highly dynamic genome with recombination between lineages that have been considered to have different associations (i.e., hospital- versus animal-associated). Trees of clades A2 and B are shown in Supplemental Figure S2.

Having identified clades A1, A2, and B, we plotted each clade against the three different culture media used for bacterial isolation (Fig. 2C). This confirmed that ampicillin-susceptible *E. faecium* resided in clades B and A2, AREfm resided in clades A2 and A1, and VREfm was exclusive to clade A1. We observed that clade A2 isolates constituted approximately half of *E. faecium* in untreated sewage. None of the plants knowingly received farm effluent, suggesting that these “animal-associated” lineages may be commonly carried by people in the community. Hospital-adapted lineages of clade A1 were present in treatment plants in both urban and rural settings and were not restricted to wastewater plants downstream from hospitals.

**Figure 3.**
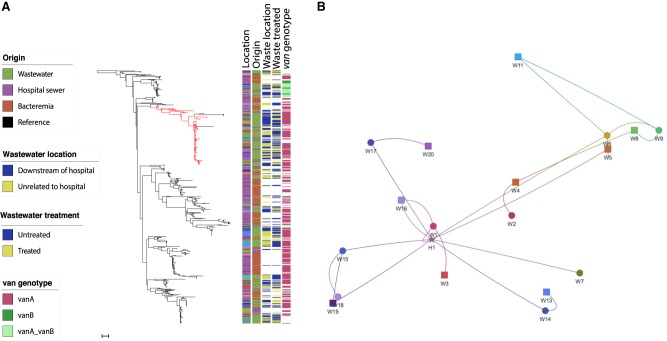
Distribution of clade A1 *E. faecium* and clonal expansion of dominant clusters. (*A*) Maximum-likelihood tree based on SNPs in the core genes of 481 clade A1 *E. faecium* isolates colored by geographical location in relation to receipt of hospital sewage, origin, wastewater plant location in relation to hospitals, treated or untreated wastewater, and presence of *van* genes. Branch leading to and including the dominant cluster is colored in red. Scale bar, ∼55 SNPs. (*B*) Phylogenetic network analysis showing relatedness of *E. faecium* based on place of origin displayed as shapes corresponding to geographical coordinates. Circles: wastewater treatment plants located downstream from hospitals; squares: wastewater treatment plants unrelated to hospital waste; star: Cambridge University Hospitals (bloodstream isolates). The edges (lines) link isolates that were very closely related (within 5 SNPs in the core genome).

**Figure 4.**
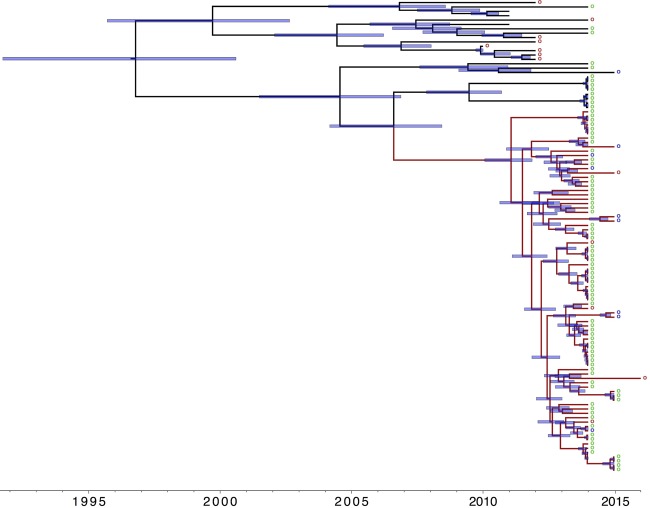
Timeline of emergence of dominant *E. faecium* cluster. Maximum clade credibility tree of branch leading to and including the dominant cluster from BEAST analysis (*n* = 108 isolates). Branch in red includes all isolates of cluster (and neighboring isolates). Blue bars indicate 95% highest probability density (HPD) values. (*Right*) Circles denote origin of isolates. Green: wastewater; purple: CUH sewage; red: bloodstream infection. Time bar (yr) shown at the *bottom*.

We then focused our analysis on the 481 isolates in clade A1 to determine the comparative phylogeny and gene content between isolates from wastewater and patients with bloodstream infection. A maximum-likelihood phylogeny following removal of recombination and mobile elements showed a diverse population interspersed with clonal expansions (Fig. 3A). Numerous clonal expansions were noted, with closely related isolates shared between two or more locations, including wastewater treatment plants (located downstream and unrelated to hospitals), hospital sewage, and/or invasive isolates. The largest cluster (termed C1) contained 72 isolates (15% of the total), including 64 from 15/20 wastewater treatment plants, four from CUH sewage, and four bloodstream isolates from CUH in 2014–15. The majority of isolates (68/72) in this cluster belonged to ST80. The substitution rate of the branch leading to this large cluster (calculated based on isolates shown in red in Fig. 3A) was 9.21 SNP/genome/yr (3.23 × 10^−6^ SNPs/site/yr), estimating the most recent common ancestor of C1 and related isolates at ∼9.4 yr (95% highest posterior density [HPD], 7.6–11.8 yr), with 83/91 (91%) having diverged 4.9 yr ago (95% HPD, 4.1–5.9 yr) (Fig. 4). This suggests their recent emergence and dissemination in our geographic region, including isolates that caused invasive disease.

The majority of wastewater treatment plants each contained a genetically diverse *E. faecium* clade A1 population based on core genome pairwise SNP distance (Supplemental Fig. S3a). This diversity was comparable to the diversity reported for isolates associated with bloodstream infection from across the United Kingdom (Raven et al. 2016) and was not affected by the treatment process (median SNP distance 251 vs. 242 in raw and treated wastewater, respectively, *P* = 0.54) (Supplemental Fig. S3b). In contrast, the minimum SNP distance between untreated and treated wastewater samples was zero for 14 of 18 evaluable locations (range 0–10), suggesting persistence of some strains at each plant following treatment (Supplemental Fig. S3c). Closely related isolates were also detected between different wastewater treatment plants (Supplemental Fig. S3d).

Comparison of relatedness between *E. faecium* from wastewater and bloodstream infection showed that the minimum SNP distance between any bloodstream to wastewater isolate did not differ significantly for plants located downstream from hospitals versus plants unrelated to hospitals (median 35 vs. 46, *P* = 0.09) (Supplemental Fig. S3e). Network analysis using a strict 5-SNP cut-off (consistent with less than 1 yr of evolution based on this study and previous estimates [Raven et al. 2017]) revealed geographical clustering of wastewater treatment plants. Bloodstream isolates from CUH formed a direct network with 9/20 plants (three unrelated to hospital waste), indicating recent dissemination of invasive and wastewater *E. faecium* across the region (Fig. 3B).

### Genes encoding antibiotic resistance and virulence

**Figure 5.**
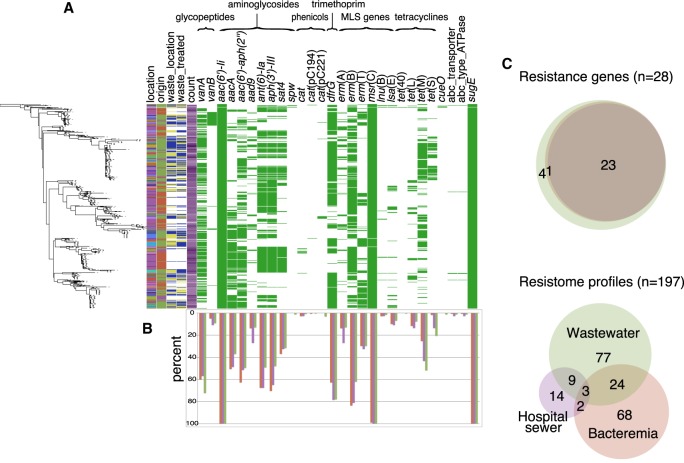
Distribution of antimicrobial resistance genes in hospital-adapted *E. faecium* from bloodstream infections, hospital, and municipal sewage. (*A*) Presence of antibiotic, metal, and biocide resistance genes grouped by antibiotic family in clade A1 *E. faecium* isolates shown against the core SNP maximum likelihood tree (*left*-hand side). Colors in *left*-hand columns correspond to those in Figure 3. Antimicrobial resistance gene columns: green, present; white, absent. (*B*) Frequency of detection of each gene in wastewater, hospital sewage, and bloodstream isolates (green: wastewater; purple: hospital sewer; red: bloodstream). (*C*) Venn diagrams showing the degree of overlap between individual and combined resistance gene profiles in the three reservoirs. (*Upper* panel) *Innermost* circle: bloodstream; *middle* circle: hospital sewer; *outer* circle: wastewater.

Having established close genetic relatedness between isolates from different reservoirs and locations based on the core genome, we analyzed the pangenome of clade A1 isolates for the presence of antibiotic, metal, and biocide resistance genes (Fig. 5A,B). Of 28 different resistance genes detected, 23 (82%) were represented in bloodstream, hospital sewage, and municipal wastewater isolates. Municipal wastewater isolates harbored the greatest diversity, including *spw*, *cat*(pC221), *tet*(40), and *cueO* that confer spectinomycin, chloramphenicol, tetracycline, and copper resistance, respectively, which were exclusive to wastewater isolates (Fig. 5C, top). There were 197 different antibiotic resistance gene combinations, with only three profiles shared between all three reservoirs and greater diversity detected in wastewater (Fig. 5C, bottom). The median number of genes detected was 9 (interquartile range [IQR] 8–10), 9 (IQR 9–11), and 8 (IQR 7–11) for bloodstream, hospital sewage, and municipal wastewater isolates, respectively. An analysis of putative virulence genes led us to detect 32 genes that were present in wastewater, hospital sewage, and bloodstream isolates (Supplemental Fig. S4). No gene was exclusively present in wastewater or bloodstream isolates based on a pangenome analysis (Supplemental Fig. S5).

**Figure 6.**
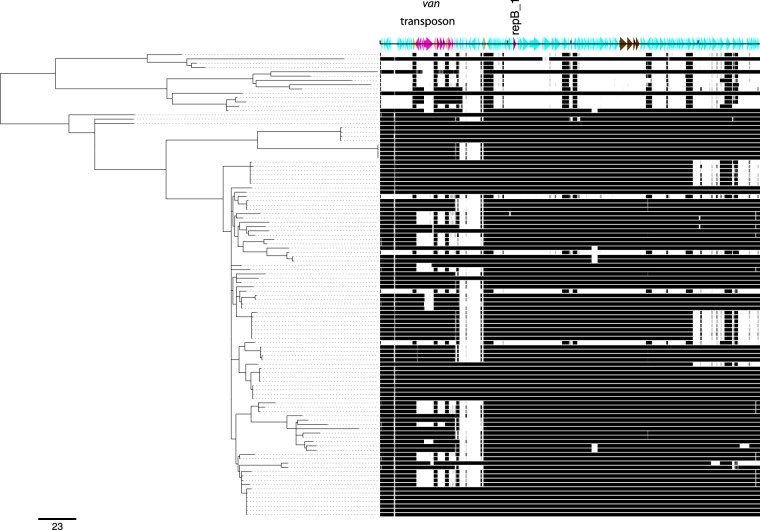
Reads mapped against *vanA* reference plasmid EC503 showing common plasmid backbone present in bloodstream and wastewater isolates. *Left*-hand side: maximum-likelihood tree of branch leading to dominant clade (*n* = 108). Scale bar, 23 SNPs. The density of the horizontal bars corresponds to the read coverage, with black indicating presence and white absence. (*Top*) Turquoise arrows, coding sequences: pink, *vanA* transposon genes; orange, IS*1216E*; purple, novel *repB_1* gene; brown, genes encoding for novel putative cell-surface proteins (Cna protein B-type domains) and associated sortases. Markings shown at 20,000 bp. Vancomycin susceptibility was associated with lack of plasmid or lack of *vanA* transposon (with or without downstream 3′ region). Full sequence and annotation files of plasmid EC503 are available in Supplemental File S2.

We investigated the genetic context of the *vanA* transposon for 108 isolates belonging to the lineage leading to the dominant cluster C1 investigated above, of which 80 contained the *vanA* gene (see Methods). Ninety-four isolates had reads mapping at >80% (65 at >90%) to a completely sequenced plasmid from a dominant clinical cluster of bloodstream isolates circulating in CUH since 2007 (Raven et al. 2017) that contained a novel *rep* gene and two genes encoding putative cell-surface proteins with Cna protein B-type domains but no additional resistance genes. Absence of *vanA* genes was associated either with loss of this plasmid or with loss of the *vanA* transposon and an approximately 9.5-kb downstream region flanked by IS*1216E* (Fig. 6).

## Discussion

This systematic genomic survey of *E. faecium* in wastewater indicates that hospital-adapted lineages of VREfm are widespread in wastewater across the East of England, adding to previous studies restricted to wastewater collected downstream from hospitals (Novais et al. 2005; Caplin et al. 2008; Leclercq et al. 2013; Varela et al. 2013). Wastewater treatment did not prevent downstream environmental contamination with these lineages, with the majority of plants releasing drug-resistant *E. faecium* into the environment. Stronger controls are required to prevent these organisms being freely released into the water system. Ultraviolet light was used at three study plants and was effective in decontaminating wastewater prior to release. This represents a ready solution that would reduce environmental contamination with VREfm and drug-resistant bacteria more generally.

The use of culture media containing incremental antibiotic selective pressure allowed us to sample the diversity of *E. faecium* and quantify and obtain sufficient numbers of resistant lineages that were present in wastewater as a minority of the overall population. The observation that AREfm and VREfm counts were higher in untreated wastewater from plants in direct receipt of hospital sewage versus those that were not is predictable and consistent with the finding that the relative proportion of VREfm in wastewater from the CUH hospital main sewer was high. However, the detection of VREfm at all treatment plants is an important finding and consistent with widespread dissemination of drug-resistant lineages in the community. Potential reservoirs include people with past or on-going healthcare contact, including hospital patients and those in long-term care facilities (Brodrick et al. 2016). Given the scale of VREfm detection, including from plants in rural areas, it is possible that VREfm carriage extends to the wider healthy population. This could include healthcare workers, family contacts of people receiving healthcare, and people with no direct or indirect healthcare-associated contact. Potential sources in the wider community include the environment and the food chain. There is a lack of data on rates of VREfm carriage by healthy populations, with recent data from our group suggesting that VREfm in the food chain differs genetically from human and wastewater VREfm (Gouliouris et al. 2018). Further systematic studies are needed to identify community carriage.

Whole-genome sequence-based comparison of *E. faecium* from wastewater and patients with significant infection is essential for the accurate determination of relatedness between bacteria from disease and nondisease reservoirs. The core genome phylogenies generated here demonstrated that these were genetically intermixed and detected numerous highly related clusters that contained isolates from patients and wastewater. Wastewater isolates from nine treatment plants (including those that did not receive hospital sewage) were closely related to *E. faecium* associated with bloodstream infection at CUH. Furthermore, isolates from patients and nonhuman sources shared the majority of antibiotic resistance and virulence genes investigated. These observations build on our culture-based findings and provide strong evidence for the widespread dissemination of highly related healthcare-associated drug-resistant *E. faecium* lineages.

In conclusion, our findings highlight the challenges of controlling healthcare-associated dissemination of VREfm. The extent to which isolates from humans and wastewater were related indicates that wastewater could be used for the surveillance of circulating VREfm lineages. The risk posed to human health from extensive release of VREfm into the environment is uncertain but could be controlled by improving wastewater decontamination both at the hospital and municipal waste level. Both the risk of release and the benefit derived from preventing this require further study.

## Methods

### Wastewater sampling and microbiology

We performed a cross-sectional survey of 20 municipal wastewater treatment plants in the East of England between June 2014 and January 2015. Ten plants directly received sewage from acute NHS Hospital Trusts (median distance between plant and respective hospital: 5.3 km [range 3.3–9.6 km] downstream), and 10 plants did not directly receive hospital sewage. Treatment plant characteristics are shown in Supplemental Table S1 and described in Supplemental Methods. In brief, all plants processed wastewater using primary treatment (sedimentation of large particles in primary settlement tanks) and secondary treatment, which consisted of activated sludge and/or biological trickling filter beds. Ten plants utilized an additional tertiary step, such as lagooning (reed beds) or sand filtration. Wastewater effluent from three plants with discharge into sensitive coastal waters (shellfish production areas) was disinfected by a terminal ultraviolet light step. Untreated and treated wastewater were obtained from each plant. Grab samples of 0.5 L were collected at each sampling point and mixed into 1 L sterile bottles containing 18 mg sodium thiosulphate (Sigma-Aldrich). Additional sampling was performed at the hospital sewer (main septic tank) of CUH, when a single 1 L wastewater sample was obtained at four spaced time points between September 2014 and December 2015.

Bacterial quantification in wastewater was achieved by the membrane filtration method adapted from previously described protocols (Figueras et al. 2000) using the EZ-Stream vacuum pump and EZ-Fit Manifold system (Merck Millipore). Membranes were placed onto Slanetz-Bartley (SB) agar (Oxoid); ampicillin-selective medium BBL Enterococcosel agar (BD) supplemented with 30 mg/L ampicillin (Sigma-Aldrich); and vancomycin-selective medium *Brilliance* VRE chromogenic agar (Oxoid). Bacteria were identified to the species level using matrix-assisted laser desorption/ionization time-of-flight mass spectrometry (MALDI-TOF MS; Biotyper version 3.1, Bruker Daltonics). Antimicrobial susceptibility testing was determined using the P607 card on the VITEK2 instrument (bioMérieux) (Supplemental Fig. S6). More details of sample processing are provided in Supplemental Methods.

### Bacteria for genome sequencing

Up to 20 colonies (isolates) were selected from primary cultures from each plant for genome sequencing (equal numbers from untreated and treated wastewater). Final selection was based on diversity of antibiotic resistance patterns (100 of the 110 possible patterns were included), which we used as a rough surrogate for genetic diversity. A further 10 isolates were selected from each CUH sewer sample using the same criteria. In all, 428 wastewater isolates were sequenced (388 from treatment plants and 40 from CUH sewers), of which five were subsequently excluded based on sequence quality metrics. Supplemental Table S2 provides the details of origin and isolation year for each isolate. The clinical isolate collection used here contained 187 *E. faecium* associated with bloodstream infection in 187 patients, as follows: 140 patients at CUH between 2010–2012, 24 patients at four further hospitals in the East of England between 2010–2012, and 23 consecutive cases of *E. faecium* bacteremia in hematology patients at CUH between May 2014 and April 2016. Isolates from 2010–2012 had already been sequenced, and data were downloaded from the Wellcome Sanger Institute. The remainder were sequenced as part of this project. DNA extraction and library preparation were performed as previously described (Raven et al. 2017). DNA libraries were sequenced using the Illumina HiSeq platform (Illumina Inc.). Further details can be found in Supplemental Methods.

### Phylogenetic analyses

A phylogenetic analysis was undertaken combining 620 genomes (423 wastewater, 187 clinical, and 10 nonstudy publicly available complete *E. faecium* genomes (see Supplemental Table S2 for accession numbers). Genomes were aligned to define a core and accessory genome using Roary (Page et al. 2015). The resulting alignment of 100,136 core single nucleotide polymorphisms (SNPs) was used to create a phylogeny with RAxML (Stamatakis 2014) and to produce a tree-independent hierarchical Bayesian clustering with hierBAPS (Cheng et al. 2013). Further details are provided in the Supplemental Methods. Multilocus sequence typing was determined from genome assemblies using MLST Check (Page et al. 2016) with novel alleles and STs deposited in the pubMLST website (https://pubmlst.org/efaecium/). Absence of the *pstS* gene in two isolates was confirmed by SRST2 (Inouye et al. 2014).

The hospital-adapted lineage (A1) was defined by phylogenetic methods (see Supplemental Methods for details) and a phylogenetic tree of 481 genomes created using RAxML based on mapping to the Aus0004 genome (ENA accession number CP003351). Pairwise SNP distances were calculated from the core genome using a custom script available as Supplemental Code, and at https://github.com/simonrharris/pairwise_difference_count. Timeline reconstruction of the largest cluster was performed using BEAST v1.8.2 (Drummond et al. 2012), as described in the Supplemental Methods.

### Analysis of antimicrobial resistance, virulence genes, and plasmids

Acquired genes encoding antimicrobial resistance were detected using a manually curated and updated version of the ResFinder database (compiled in 2012) (Zankari et al. 2012; additional genes are listed in Supplemental Table S4). A database of putative virulence genes was created based on *E. faecium* genes from the Virulence Factor Database (VFDB) (http://www.mgc.ac.cn/cgi-bin/VFs/genus.cgi?Genus=Enterococcus) (Chen et al. 2016) and supplemented after a literature review (Supplemental Table S5). The raw genomic reads were queried for resistance and virulence genes using Antibiotic Resistance Identification by Assembly (ARIBA) v2.5.0 (https://github.com/sanger-pathogens/ariba) (Hunt et al. 2017) with default length and match thresholds of 95% and 90%, respectively. Further processing of the output data is described in the Supplemental Methods. Plasmid analysis was performed using plasmidSPAdes (Antipov et al. 2016) and BLASTN, with details provided in the Supplemental Methods.

Statistical analyses were performed using STATA v13.1 (StataCorp LLC), as described in Supplemental Methods.

### Ethical approval

The study protocol was approved by the CUH Research and Development Department (ref: A093285) and the National Research Ethics Service East of England Ethics Committee (ref: 12/EE/0439 and 14/EE/1123).

## Data access

Sequence data from this study have been submitted to the European Nucleotide Archive (ENA; www.ebi.ac.uk/ena) under the accession numbers listed in Supplemental Table S2. The MATLAB script described in Supplemental Methods is available in Supplemental File S1.

## Competing interest statement

J.P. and S.J.P. are paid consultants to Next Gen Diagnostics. S.J.P. is a consultant to Specific.

## Supplementary Material

Supplemental Material
