# Supplementary material for: Detection of vancomycin-resistant Enterococcus faecium hospital-adapted lineages in municipal wastewater treatment plants indicates widespread distribution and release into the environment
Source: Genome Res. 2019 Apr;29(4):626–34. doi: 10.1101/gr.232629.117 (PMC6442392; doi:10.1101/gr.232629.117)
Supplement: Supplemental Material [file supp_29_4_626__index.html]

Detection of vancomycin-resistant Enterococcus faecium hospital-adapted lineages in municipal wastewater treatment plants indicates widespread distribution and release into the environment — Supplemental Material 

# Detection of vancomycin-resistant *Enterococcus faecium* hospital-adapted lineages in municipal wastewater treatment plants indicates widespread distribution and release into the environment

## Supplemental Material

- Supplemental\_Material\_Final.docx
- Supplemental\_File\_S1.rtf
- Supplemental\_File\_S2.zip
- Supplemental\_Table\_S2.xlsx
- Supplemental\_Table\_S3.xlsx
- Supplemental\_Table\_S4.xlsx
- Supplemental\_Table\_S5.xlsx
