# Supplementary material for: Detection of vancomycin-resistant Enterococcus faecium hospital-adapted lineages in municipal wastewater treatment plants indicates widespread distribution and release into the environment
Source: Genome Res. 2019 Apr;29(4):626–34. doi: 10.1101/gr.232629.117 (PMC6442392; doi:10.1101/gr.232629.117)
Supplement: Supplemental Material [file supp_gr.232629.117_Supplemental_Material_.docx]

**Supplemental material to: Detection of vancomycin-resistant *Enterococcus faecium* hospital-adapted lineages in municipal wastewater treatment plants indicates widespread distribution and release into the environment**

Theodore Gouliouris, Kathy E. Raven, Danesh Moradigaravand, Catherine Ludden, Francesc Coll, Beth Blane, Plamena Naydenova, Carolyne Horner, Nicholas M. Brown, Jukka Corander, Direk Limmathurotsakul, Julian Parkhill, Sharon J. Peacock

**Contents:**

**Supplemental Methods**

**Supplemental Table S1**

**Supplemental Figure S1**

**Supplemental Figure S2**

**Supplemental Figure S3**

**Supplemental Figure S4**

**Supplemental Figure S5**

**Supplemental Figure S6**

**Supplemental Methods**

**Characteristics of wastewater plants**

The plants processed a dry weather flow of between 5 and 505 litres per second (L/s), with a peak capacity (under storm conditions) of between 5 and 1400 L/s. Plants were characterized based on population equivalent (PE) and whether they were urban or rural. One PE was defined as the organic biodegradable load having a 5-day biochemical oxygen demand (BOD5) of 60 g of oxygen per day (Department for Environment, Food and Rural Affairs 2012). The PE ranged from 1,567 to 325,000 for a combined PE of ca. 1,639,000 (representing 2.4% of the UK total PE based on 2010 figures) (Department for Environment, Food and Rural Affairs 2012). The distinction between urban and rural agglomerations served by the wastewater plants was based on the UK Department for Environment, Food & Rural Affairs Rural Affairs (DEFRA) rural-urban classification defining urban areas as those with more than 10,000 residents (<https://www.gov.uk/government/statistics/2011-rural-urban-classification>). Influent (incoming sewage) was predominantly of human residential origin delivered via a combined sewer system (i.e. including both domestic waste and rainwater), no regular farm inflow, and additional industrial waste received in plants that were located in larger urban centres. All plants processed wastewater using primary treatment (sedimentation of large particles in primary settlement tanks) and secondary treatment, which consisted of activated sludge and/or biological trickling filter beds. Ten plants utilized an additional tertiary step, such as lagooning (reed beds) or sand filtration. Wastewater effluent from 3 plants with discharge into sensitive coastal waters (shellfish production areas) was disinfected by a terminal ultraviolet light step.

**Wastewater processing and bacterial identification**

Treated wastewater was always obtained first, from designate sampling points prior to discharge onto surface waters. Untreated wastewater was obtained approximately 15 minutes later, after the initial screening process that removes large solids but prior to reaching the primary settlement tanks. All samples were transported to the laboratory on ice packs in the dark (transit time 30 min to 3 hours) and refrigerated at 4°C on arrival to the laboratory. Processing was completed within 12 hours for all samples (within 6 hours for 15/20 samples). Bacterial quantification was achieved by the membrane filtration method adapted from previously described protocols (Figueras et al. 2000) using the EZ-Stream vacuum pump and EZ-Fit^TM^ Manifold system (Merck Millipore, Darmstadt, Germany). One mL of triplicate serial ten-fold dilutions, 10 mL of treated and untreated wastewater and 100 mL of treated wastewater samples were concentrated using the filtration technique onto 0.45 μm pore size, mixed cellulose ester filter membranes (S-Pak, Merck Millipore, Darmstadt, Germany). Membranes were then placed onto the surface of a range of selective plates representing increasing levels of antibiotic selective pressure and incubated for 48 h at 37°C in air. The plates consisted of medium with no selection for antibiotic resistance (Slanetz-Bartley (SB) agar (Oxoid, Basingstoke, UK)); ampicillin-selective medium BBL^TM^ Enterococcosel^TM^ agar (BD, Oxford, UK) supplemented with 30 mg/L ampicillin (Sigma-Aldrich, Poole, UK); and vancomycin-selective medium *Brilliance*^TM^ VRE chromogenic agar (Oxoid, Basingstoke, UK). Enumeration of colony forming units (cfu) with morphology consistent with *E. faecium* was performed on plates containing 10-80 colonies and were expressed as log_10_ (mean cfu/100 ml + 1). Counts of presumptively identified organisms were revised as necessary following definitive identification of organisms (see below).

At least one isolate of each bacterial colony morphology type suspected to be *Enterococcus* sp. was picked from every agar plate and speciated using matrix-assisted laser desorption/ionization time-of-flight mass spectrometry (MALDI-TOF MS; Biotyper version 3.1, Bruker Daltonics, Coventry, UK). Antimicrobial susceptibility testing was determined using the P607 card on the VITEK®2 instrument (bioMérieux, Marcy l’Étoile, France). For isolates with discrepant Van phenotype and *van* genotype results, MIC determination by MIC strip gradient testing using ETEST® strips (bioMérieux, Marcy l’Étoile, France) was performed according to the manufacturer’s instructions. Isolates were stored at -80°C on glycerol beads. Phenotypic testing showed that 330/345 (44%) colonies picked from non-selective plates (median 8, IQR 8-10 colonies picked per sample) were *E. faecium* (n=153) or other *Enterococcus* spp. (n=177), and that 136/138 (99%) of colonies further tested were ampicillin-susceptible *E. faecium*. 175 of 178 (98%) colonies picked from ampicillin-resistance media (median 5, IQR 4-6 colonies picked per sample) were AREfm, and 248/250 (99%) enterococci picked from vancomycin selective media (median 6, IQR 6-10 colonies picked per sample) were ampicillin-resistant VREfm (the remaining two were vancomycin-resistant *E. faecalis*). Antibiotic resistance profiles of 529 *E. faecium* isolates (137 from non-selective plates, 163 from ampicillin-selective plates and 229 from vancomycin-selective plates) are shown in Supplemental Figure S6.

**Illumina sequencing, genome assembly and annotation**

Sequencing was performed with 100 or 125-cycle paired-end reads to give a mean coverage of 132 reads per nucleotide (range 47-333). *De novo* multi-contig draft assemblies were generated using a published pipeline (Page et al. 2016). Automated annotation of draft genomes and 10 publically available complete genomes (see below) was performed using Prokka v1.11 (Seemann 2014) and a genus specific database from RefSeq. The assembly pipeline generated an average total length of 2,934,268 bp (range 2,428,095– 3,274,556) from 129 contigs (range 9-233) with an average contig length of 30,381 bp (range 13,587 – 281,694 bp) and an N50 of 79,364 bp (range 29,117-1,321,638 bp).

**Phylogeny based on shared core genes using Roary, pangenome and hierarchical Bayesian clustering**

Roary was run using a BLASTP identity of 90% and core definition of 99%. We identified a total of 15,733 predicted coding sequences, with 1,336 core (present in 99% of isolates) and 14,397 accessory genes (variably present). The resulting alignment of 100,136 core single nucleotide polymorphisms (SNPs) (identified using snp-sites) (Page et al. 2016) out of 1,174,835 positions was used to infer a RaxML (Stamatakis 2014) phylogeny with 100 bootstraps and a midpoint root using a general time reversible (GTR) evolutionary model and a gamma correction for among site rate variation. hierBAPS was run following exclusion of 15,061 singleton SNPs for which the minor allele was present only in a single isolate within the population using a custom-made Matlab script (available as Supplemental File S1). The hierBAPS software was run with five replicates of the posterior maximization algorithm using prior upper bounds of 200-500 clusters. All runs resulted in the same maximum *a posteriori* partition estimate, indicating high peakedness of the posterior distribution over the clustering space.

**Phylogeny based on core genome mapping to Aus0004**

The hospital-adapted lineage (A1) was defined phylogenetically in a stepwise manner. First, *E. faecium* genomes belonging to the distant clade B (n=42) and the most basal part of clade A2 of paraphyletic BAPS-4 were excluded (n=37). Sequence reads from the remaining 531 isolates were mapped to Aus0004 genome (ENA accession number CP003351) using SMALT v0.7.4 (<https://sourceforge.net/projects/smalt/>) as previously described (Moradigaravand et al. 2017). Mobile genetic elements identified using PHASTer (Arndt et al. 2016), ISFinder (Siguier 2006) and annotation, and recombination events using Gubbins (Croucher et al. 2014) were removed to define the core genome. A midpoint-rooted maximum likelihood tree was created using RaxML (Stamatakis 2014) and 100 bootstraps. BAPS group 4 and related BAPS-2 isolates were assigned to clade A2.2 and were distinguished from the remaining 481 isolates which were assigned to clade A1 and correspond to STs characteristic of CC17. Gubbins was repeated for the 481 clade A1 isolates using the closest excluded isolate (belonging to ST192) for rooting and 100 bootstraps.

**Timeline reconstruction of dominant *E. faecium* cluster (C1)**

The lineage containing the largest cluster was defined by including neighboring isolates belonging to second order BAPS groups 1-1 and 1-4 (n=105). We searched for other closely related isolates by interrogating retrospective datasets of bloodstream isolates from the East of England and the rest of the UK and Ireland (2001-2012) (Raven et al. 2016) and an international collection of isolates published by Lebreton *et al*. (Lebreton et al. 2013) by running a core genome tree in Roary. From these, we identified only 3 additional closely related isolates from 2011 from London (n=1) and the South West (n=2). The collection of 108 isolates was mapped using SMALT against a local reference chosen amongst the isolates from the studied cluster with the lowest contig number. Mobile genetic elements and recombination were removed as before. In addition, putative plasmid sequences were identified using BLASTN (blast.ncbi.nlm.nih.gov) and removed along with contigs less than 500 bp in length. BEAST v1.8.2 (Drummond et al. 2012) was used to determine the clock rate and the time to the most recent common ancestor. We used strict, relaxed log-normal and relaxed exponential clock models each combined with constant, exponential and skyline population models. Each combination was computed in triplicate for 100 million iterations and sampled every 1,000 generations. Models that failed to converge based on visual inspection or had effective sampling size (ESS) values <200 for key parameters were discarded. Only the strict clock with constant tree model always converged and this model was used to estimate the clock rate and the time to most recent common ancestor (MRCA). We excluded 10% of trees as burn-in and generated a maximum clade credibility tree using TreeAnnotator (part of the BEAST package) to summarize the results and obtain confidence intervals for node ages.

**Analysis of antimicrobial resistance and virulence genes**

For genes that ARIBA reported as fragmented, the query sequence was checked by BLASTN against the respective genome assembly and defined as present if a length of 100% and sequence match of 90% were detected. Additional manual checks were implemented for genes coding for microbial surface components recognising adhesive matrix molecules (MSCRAMMS) that contain LPxTG or LPxTG-like motifs reported as fragmented by ARIBA due to the presence of internal repeat elements. Fragmented genes were manually identified in the whole genome assemblies and annotated as present if the coding regions for the N terminus signal peptide and the LPxTG motifs were present and the amino acid sequences could be aligned using SeaView (<http://doua.prabi.fr/software/seaview>) showing variations in length due to different numbers of internal repeats. For sequences that were fragmented even in the whole genome assembly (often present at the ends of two different contigs), we reported them as fragmented. For gene *prpA*, we noted a dominant variant present in 440 sequences that lacked the LPKSG (LPxTG-like) motif, which we labelled as *prpA*-variant.

**Plasmid analysis**

Putative plasmid sequences were identified for the 108 isolates studied above (under timeline reconstruction) using plasmidSPAdes (Antipov et al. 2016). *rep* typing of putative plasmid contigs was performed by BLASTN using the PlasmidFinder Gram positive database available at <https://cge.cbs.dtu.dk/services/PlasmidFinder/> and downloaded on 08.03.17. Confirmation of presence of the *vanA*, *vanS*, and *vanZ* genes on putative plasmid contigs was obtained by BLASTN using 95% and 90% sequence length and match thresholds, respectively. These three genes were chosen as representative of the *vanA* transposon as they are usually assembled on three separate contigs due to interspersed IS elements. Putative plasmid contigs were compared to the ncbi database and to a local collection of fully sequenced *E. faecium* plasmids (Raven et al. 2017). The best match was a plasmid previously reported in (Raven et al. 2017), the sequence and annotation files of which are available in Supplemental File S2. Genomic reads were mapped against this plasmid using SMALT v0.7.4 as above and visualized using an in-house tool.

**Statistical analysis and data visualization**

Continuous variables are presented as medians, and interquartile ranges (IQRs) are presented in terms of 25th and 75th percentiles. Comparisons of unpaired continuous variables were performed using the Student *t*-test and ANOVA. Comparisons of paired continuous variables were performed using the paired *t*-test, with analysis of covariance (ANCOVA) also used to evaluate the effect of water treatment. Sensitivity analyses were performed using non-parametric tests including Mann-Whitney test, Kruskal-Wallis test, and Wilcoxon signed-rank test when appropriate. All tests were two-sided and α=0.05 was used for statistical significance. Visualization of phylogenetic trees was performed in iTOL v3 (Letunic and Bork 2016), FigTree v1.4.2 (<http://tree.bio.ed.ac.uk/software/figtree/>) and Phandango (<https://jameshadfield.github.io/phandango/>) (Hadfield et al. 2017). Networks of closely related isolates using a 5-SNP cut-off were visualized in Microreact (<https://microreact.org/>). Graphs were produced in Stata and R.

**REFERENCES**

Antipov D, Hartwick N, Shen M, Raiko M, Lapidus A, Pevzner PA. 2016. plasmidSPAdes: assembling plasmids from whole genome sequencing data. *Bioinformatics* **32**

Arndt D, Grant JR, Marcu A, Sajed T, Pon A, Liang Y, Wishart DS. 2016. PHASTER: a better, faster version of the PHAST phage search tool. *Nucleic Acids Res* **44**: W16–W21.

Croucher NJ, Page AJ, Connor TR, Delaney AJ, Keane JA, Bentley SD, Parkhill J, Harris SR. 2014. Rapid phylogenetic analysis of large samples of recombinant bacterial whole genome sequences using Gubbins. *Nucleic Acids Res* **43**: e15.

Department for Environment, Food and Rural Affairs. 2012. *Waste water treatment in the United Kingdom*.

Drummond AJ, Suchard MA, Xie D, Rambaut A. 2012. Bayesian phylogenetics with BEAUti and the BEAST 1.7. *Mol Biol Evol* **29**: 1969–73.

Figueras MJ, Borrego J, Pike EB, Robertson W, Ashbolt N. 2000. Sanitary inspection and microbiological water quality*. In Monitoring Bathing Waters - A practical guide tot he design and implementation of assessments and monitoring programmes*. (eds. J. Bartram and G. Rees). World Health Organisation, New York.

Hadfield J, Croucher NJ, Goater RJ, Abudahab K, Aanensen DM, Harris SR. 2017. Phandango: an interactive viewer for bacterial population genomics. *Bioinformatics* 1–2.

Lebreton F, van Schaik W, Mcguire AM, Godfrey P, Griggs A, Corander J, Cheng L, Saif S, Young S, Zeng Q, et al. 2013. Emergence of epidemic multidrug-resistant *Enterococcus faecium* from animal and commensal strains. *MBio* **4**: 1–9.

Letunic I, Bork P. 2016. Interactive tree of life (iTOL) v3: an online tool for the display and annotation of phylogenetic and other trees. *Nucleic Acids Res* **44**: W242–W245.

Moradigaravand D, Martin V, Peacock SJ, Parkhill J. 2017. Evolution and epidemiology of multidrug-resistant Klebsiella pneumoniae in the United Kingdom and Ireland. *MBio* **8**: 1–13.

Page AJ, Taylor B, Delaney AJ, Soares J, Seeman T, Keane JA, Harris SR. 2016. SNP-sites : rapid efficient extraction of SNPs from multi-FASTA alignments. *Microb Genom***.2**: e000056.

Page AJ, De Silva N, Hunt M, Quail MA, Parkhill J, Harris SR, Otto TD, Keane JA. 2016. Robust high-throughput prokaryote de novo assembly and improvement pipeline for Illumina data. *Microb Genomics* **2**: 1–7.

Raven KE, Gouliouris T, Brodrick H, Coll F, Brown NM, Reynolds R, Reuter S, Török ME, Parkhill J, Peacock SJ. 2017. Complex Routes of Nosocomial Vancomycin-Resistant *Enterococcus faecium* Transmission Revealed by Genome Sequencing. *Clin Infect Dis* **64**: 15–17.

Raven KE, Reuter S, Reynolds R, Brodrick HJ, Russell JE, Török ME, Parkhill J, Peacock SJ. 2016. A decade of genomic history for healthcare-associated *Enterococcus faecium* in the United Kingdom and Ireland. *Genome Res* **26**: 1388–1396.

Seemann T. 2014. Prokka: Rapid prokaryotic genome annotation. *Bioinformatics* **30**: 2068–2069.

Siguier P. 2006. ISfinder: the reference centre for bacterial insertion sequences. *Nucleic Acids Res* **34**: D32–D36.

Stamatakis A. 2014. RAxML version 8: A tool for phylogenetic analysis and post-analysis of large phylogenies. *Bioinformatics* **30**: 1312–1313.

**Supplemental Table S1 Characteristics of wastewater treatment plants.**

PE: population equivalent, defined as the organic biodegradable load having a 5-day biochemical oxygen demand (BOD5) of 60 g of oxygen per day. Treatment details: Secondary: AS: activated sludge, TF: trickling filters. Tertiary: BWB: biological waterbeds; lagoon; nitrifying filter; RF: reed filters; SF: sand filters.

| **Plant** | **Date Sampled** | **Downstream of hospital** | **Urban or rural** | **PE** | **Dry flow (L/s)** | **Maximum flow (L/s)** | **Highest level of treatment** | **Details of treatment** |
| --- | --- | --- | --- | --- | --- | --- | --- | --- |
| 1 | 24/06/14 | Yes | Urban | 140867 | 432 | 1273 | Tertiary | AS, TF, SF |
| 2 | 17/07/14 | Yes | Urban | 91577 | 149 | 1044 | Tertiary | TF, SF |
| 3 | 22/07/14 | No | Urban | 16462 | 43 | 110 | Tertiary | TF, nitrifying filter |
| 4 | 12/08/14 | No | Urban | 30915 | 85 | 508 | Secondary | TF |
| 5 | 26/08/14 | No | Rural | 5000 | 11 | 32 | Tertiary | TF, lagoon |
| 6 | 26/08/14 | Yes | Urban | 325000 | 770 | 2310 | Secondary | AS |
| 7 | 01/09/14 | Yes | Urban | 135000 | 300 | 1039 | Secondary | AS |
| 8 | 22/09/14 | No | Rural | 4000 | 8 | 34 | Tertiary | AS, RF |
| 9 | 22/09/14 | Yes | Urban | 170000 | 340 | 570 | Secondary | AS |
| 10 | 25/09/14 | No | Rural | 3146 | 13 | 34 | UV light | UV |
| 11 | 25/09/14 | No | Rural | 4794 | 9 | 23 | UV light | UV |
| 12 | 10/10/14 | Yes | Urban | 78423 | 250 | 750 | Secondary | AS |
| 13 | 24/10/14 | No | Rural | 1567 | 5 | 5 | Tertiary | TF, RF |
| 14 | 24/10/14 | Yes | Urban | 127000 | 339 | 884 | UV light | UV |
| 15 | 31/10/14 | Yes | Urban | 196620 | 405 | 1278 | Secondary | AS |
| 16 | 13/11/14 | No | Urban | 16105 | 49 | 132 | Tertiary | TF, SF/RF |
| 17 | 10/12/14 | Yes | Urban | 192450 | 505 | 1400 | Tertiary | AS, BWB |
| 18 | 12/12/14 | Yes | Urban | 10261 | 173 | 173 | Tertiary | AS, BWB |
| 19 | 12/12/14 | No | Urban | 65000 | 197 | 462 | Tertiary | AS, BWB |
| 20 | 23/01/15 | No | Urban | 25000 | 75 | 500 | Secondary | TF |

**
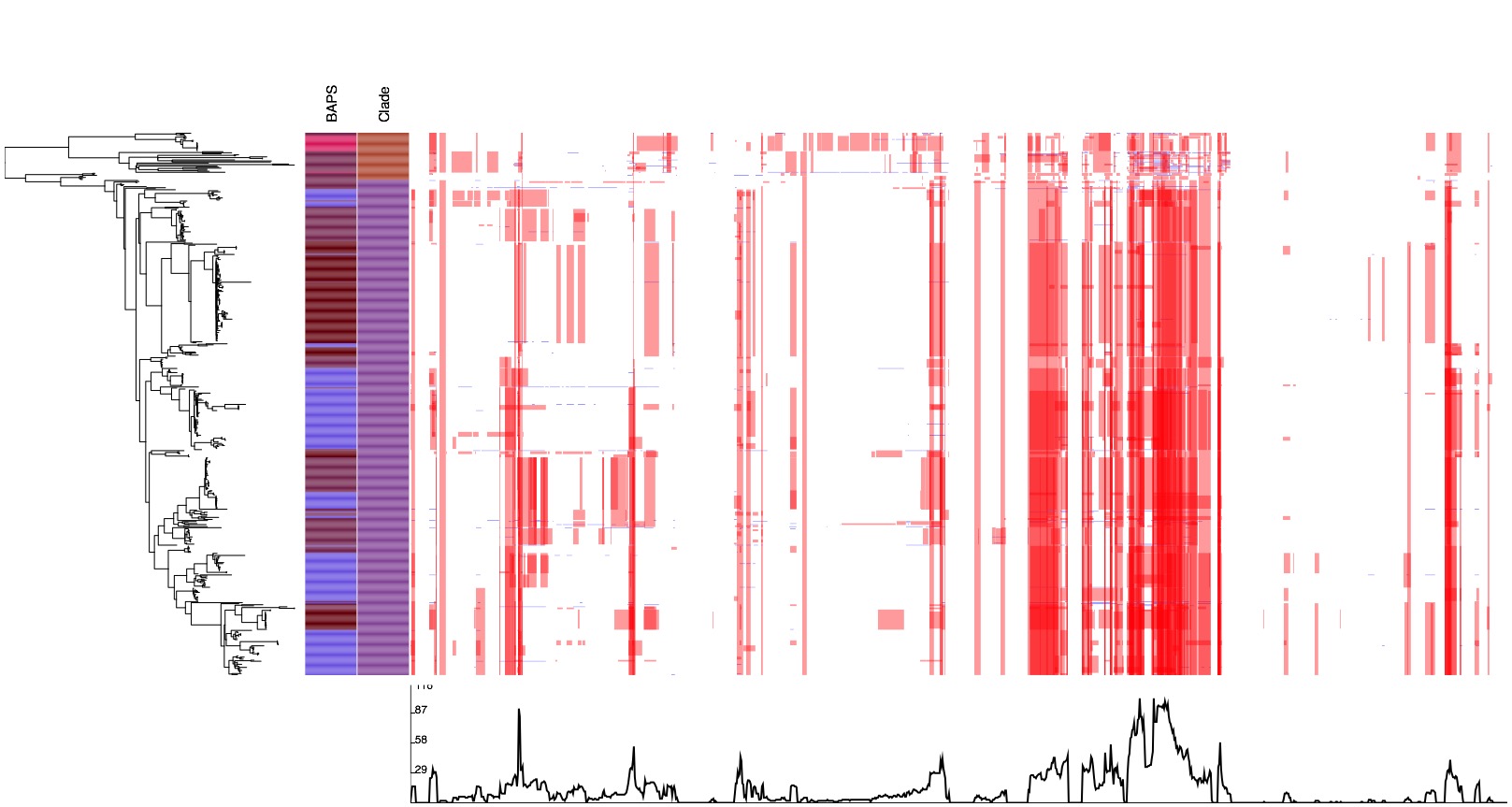
**

**Supplemental Figure S1 Predicted recombination events and phylogeny distinguishing clades A2.2 and A1.** Left: midpoint rooted maximum likelihood tree (100 bootstraps) tree of 531 isolates belonging to BAPS groups 1-4 (excluding the most basal BAPS-4 group). Recombination events are shown in red bars if present in two isolates or more, and blue if unique to a single isolate.

**Supplemental Figure S2 Phylogenetic trees of clade B and A2 isolates.** Left hand panels: Midpoint rooted maximum likelihood trees of *E. faecium* isolates belonging to (a) clade B (n=42; 41 from municipal wastewater and one from the hospital sewer) and (b) clade A2 (n=87; 80 from municipal wastewater, two from the hospital sewer and 5 bacteremia isolates). Colors in first 4 columns correspond to those in Figure 3; colors in the 5^th^ column correspond to the number of antimicrobial resistance genes detected (ranging in increasing order from dark blue to yellow). Antimicrobial resistance gene columns: Green, present; white, absent. Clades A2 and B lacked *van* genes, with a total of 6 and 30 resistance genes detected, respectively. Genes absent from clade A1 isolates consisted of *aph(2”)-Id*, *bcrA*, *erm*(A)-like, *fexA*, *lnu*(A), *optrA* and were found only in clade A2 isolates; the copper resistance gene *tcrB* was detected in both clade A2 and B. Isolates of clade B had a median of 3 resistance genes (IQR 2-4), and clade A2 4 (IQR 3-5) (cf. clade A1 median 9, IQR 7-10).

**Supplemental Figure S3 Pairwise single nucleotide polymorphism (SNP) distance of 481 clade A1 *E. faecium* isolates.** Median pairwise SNP distances shown for **a**, Within wastewater-plant diversity **b**, Diversity within all untreated (raw) and treated wastewater samples. Minimum SNP distance comparisons shown for: **c**, 20 wastewater plants before and after treatment **d**, Between wastewater plants **e**, All bloodstream infection isolates against any wastewater plant, according to being downstream or unrelated to hospital waste.

**Supplemental Figure S4 Presence of putative virulence genes in 481 clade A1 *E. faecium* isolates. _­­­­­_**Left: core SNP maximum-likelihood tree. Colors in left-hand columns correspond to those in Figure 3. Putative virulence gene columns: Green, present; light green, fragmented; dark green (*prpA*), gene variant lacking LPKSG (LPxTG-like) motif; white, absent.

**Supplemental Figure S5 Pangenome analysis of clade A1 *E. faecium* isolates from bloodstream infections (n=182) and wastewater (n=263).** Matrix shows absolute numbers of genes (counts color-coded in bar on the right) arranged according to relative frequency in each population (x and y axis numbers).

**
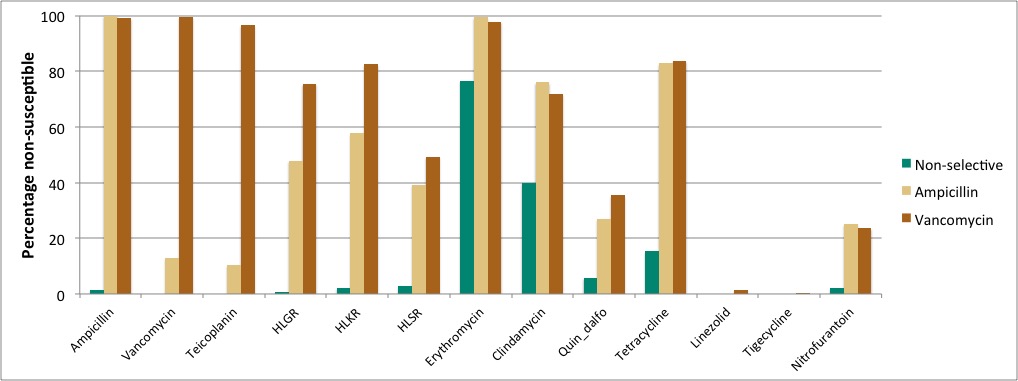
**

**Supplemental Figure S6 Susceptibility results for 529 *E. faecium* isolates recovered from non-selective (n=137), ampicillin-selective (n=163) and vancomycin-selective media (n=229).** HLGR: high-level gentamicin resistance. HLKR: high-level kanamycin resistance. HLSR: high-level streptomycin resistance. Quin_dalfo: quinupristin-dalfopristin.
