# Supplementary material for: Detection of vancomycin-resistant Enterococcus faecium hospital-adapted lineages in municipal wastewater treatment plants indicates widespread distribution and release into the environment
Source: Genome Res. 2019 Apr;29(4):626–34. doi: 10.1101/gr.232629.117 (PMC6442392; doi:10.1101/gr.232629.117)
Supplement: Supplemental Material [file supp_gr.232629.117_Supplemental_File_S1.rtf]

load results.mat%output file from hierBAPS analysis of the original alignment _loci=length(c.snpPosition); ing_locus_freqs=zeros(1,n_loci); or i=1:n_loci    freq_table=tabulate(c.snpData(:,i));    here=freq_table(:,1)<16;    if length(find(freq_table(here,2)))==2 & length(find(freq_table(here,2)==1))==1        sing_locus_freqs(1,i)=1;    end nd ingleton_loci=c.snpPosition(find(sing_locus_freqs));      or i=1:620 Header,Sequence] = fastaread('snp.aln','Blockread',i); equence(singleton_loci)='-'; if length(find(strcmp(Header,outliers)))==0 astawrite('snpfinal_nosingletonsnps.fas',Header,Sequence); endend
